# Exploration of shared gene signatures and molecular mechanisms between type 2 diabetes and osteoporosis

**DOI:** 10.1111/jcmm.18141

**Published:** 2024-05-14

**Authors:** Ashuai Du, Rong Xu, Qinglong Yang, Yingxue Lu, Xinhua Luo

**Affiliations:** ^1^ Department of Infectious Diseases Guizhou Provincial People's Hospital Guiyang China; ^2^ Department of Pathology The First People's Hospital of Changde City Changde China; ^3^ Department of General Surgery Guizhou Provincial People's Hospital Guiyang China

**Keywords:** osteoporosis, PDIA6, SLC16A1, type 2 diabetes, WGCNA

## Abstract

Type 2 diabetes mellitus (T2D) and osteoporosis (OP) are systemic metabolic diseases and often coexist. The mechanism underlying this interrelationship remains unclear. We downloaded microarray data for T2D and OP from the Gene Expression Omnibus (GEO) database. Using weighted gene co‐expression network analysis (WGCNA), we identified co‐expression modules linked to both T2D and OP. To further investigate the functional implications of these associated genes, we evaluated enrichment using ClueGO software. Additionally, we performed a biological process analysis of the genes unique in T2D and OP. We constructed a comprehensive miRNA–mRNA network by incorporating target genes and overlapping genes from the shared pool. Through the implementation of WGCNA, we successfully identified four modules that propose a plausible model that elucidates the disease pathway based on the associated and distinct gene profiles of T2D and OP. The miRNA–mRNA network analysis revealed co‐expression of *PDIA6* and *SLC16A1*; their expression was upregulated in patients with T2D and islet β‐cell lines. Remarkably, *PDIA6* and *SLC16A1* were observed to inhibit the proliferation of pancreatic β cells and promote apoptosis in vitro, while downregulation of *PDIA6* and *SLC16A1* expression led to enhanced insulin secretion. This is the first study to reveal the significant roles of *PDIA6* and *SLC16A1* in the pathogenesis of T2D and OP, thereby identifying additional genes that hold potential as indicators or targets for therapy.

## 1 INTRODUCTION

Type 2 diabetes mellitus (T2D) is a chronic ailment that may lead to severe complications, such as nephropathy, cardiovascular disease, neuropathy and retinopathy.^1^ Clinical and epidemiological evidence has established an association between T2D and osteoporosis (OP).^2^ Notably, T2D and OP commonly coexist and are both influenced by aging.^3^ Research indicates that 20%–60% of patients with T2D develop OP.^4^ The diagnostic criteria for OP rely upon identification of reduced bone mineral density (BMD) and/or susceptibility to fragility fractures.^5^ OP is a general skeletal condition characterized by diminished bone density and reduced bone microarchitecture, which increase bone frailty and vulnerability to fracture. It is noteworthy that patients with T2D present with normal or elevated BMD, and extensive prospective observations have substantiated that this malady precipitates increased bone weakness and a high likelihood of breakage.^6^, ^7^, ^8^ Furthermore, compared to the general population, patients with T2D experience augmented morbidity and mortality subsequent to fragility fractures.

With the advanced development of gene microarray technology, researchers can rapidly examine the expression data of multiple genes associated with various diseases. Weighted‐gene co‐expression network analysis (WGCNA) is a popular bioinformatics approach that effectively analyses complex interactions between genes and phenotypes.^9^ WGCNA allows organization of genes into co‐expression modules, thereby establishing critical relationships between sample features and changes in gene expression. We sought to use WGCNA to identify gene clusters, including shared genes that showed correlations and linkages between OP and T2D. This methodology has been effectively tested across a variety of biological situations, allowing for the identification of common‐risk genes and pathways linked to various disease phenotypes.

In this study, we aimed to identify co‐expression modules and genes related to T2D and OP using the extensive and rich data in the Gene Expression Omnibus (GEO) database. WGCNA was used to construct a comprehensive gene co‐expression network, which allowed us to recognize significant modules and critical genes. To the best of our knowledge, this is a first‐of‐its‐kind study to identify the shared gene signature between T2D and OP through a systems biology approach.

## 2 MATERIALS AND METHODS

### 2.1 Data set download and process

We used the terms “type 2 diabetes” and “osteoporosis” to search the GEO database for gene expression profiles related to T2D and OP. For data set selection, the following criteria were used: First, gene expression profiles that encompassed both case and control groups were selected. Second, the minimum sample size within each group was set to 10 to assure the precision of the subsequent network analysis. Third, only data sets that consisted of processed or raw data suitable for reanalysis were considered. Gene expression profiles underwent log2 transformation, and the probes were mapped to gene symbols using the annotation files in the corresponding platform.

### 2.2 Differentially expressed gene (DEG) analysis

We additionally performed DEG analysis on T2D and OP data sets to validate the presence of shared and unique genes in the two conditions. The ‘limma’ R package was used to identify DEGs, with a cut‐off of |log2(fold change)| > 0.589 and a *p* value < 0.05. The Venn tool was selected to identify overlapping DEGs between the T2D and OP data sets.^10^ We used hierarchical clustering heatmaps to visualize the patterns of these DEGs and conducted a functional enrichment analysis.

### 2.3 WGCNA procedure

WGCNA was used to generate modules related to T2D and OP. We began by selecting approximately 5000 genes with significant variation based on their variance. The WGCNA R package was used to construct the co‐expression network. Before the analysis, we performed hierarchical clustering using the Hclust function in R to identify and exclude outlier data. First, the samples were clustered to identify probable outliers. Second, the co‐expression network was built using an autonomous network construction algorithm. The R function ‘pickSoft Threshold’ was used to calculate the soft threshold power β, and adjacency was calculated by increasing co‐expression similarity to the computed threshold. Third, hierarchical clustering and dynamic tree cutting functions were used to detect modules. Fourth, gene importance and module membership were calculated to establish links between the modules and clinical characteristics. The relevant gene information for each module was retrieved for further analysis. Finally, the eigengene network was visualized.

### 2.4 Construction of miRNA–mRNA and protein–protein interaction (PPI) networks

The StarBase database was used to investigate correlations between miRNAs and mRNAs.^11^ The miRNA–mRNA regulation network was constructed based on the overlap between target genes and shared genes in T2D and OP. Furthermore, genes identified in WGCNA and DEGs in patients and healthy controls were queried in the STRING database to detect interaction and hub genes.^12^ Cytoscape was used to select and visualize hub genes and core modules, which include protein groupings that displayed the greatest amount of interaction within the PPI network.

### 2.5 Functional enrichment analysis

Genes from the modules of interest were isolated for further functional enrichment analysis. To explore functional characteristics, Gene Ontology (GO) analysis, which includes the categories of biological process (BP), cellular component (CC) and molecular function (MF), as well as the Kyoto Encyclopedia of Genes and Genomes (KEGG) pathway analysis were performed.^13^

### 2.6 Clinical sample collection

This study was approved by the Ethics Committee of Guizhou Provincial People's Hospital (Guizhou, China) and both patients and healthy volunteers provided written informed consent prior to participation. A total of 120 participants were enrolled: 60 patients with T2D and OP (T2DOP) who had not previously received treatment and 60 healthy volunteers. The patients were hospitalized at our hospital between 2022 and 2023. Blood samples were collected and stored at −80°C. The Accu‐Chek glucometer from Roche Diagnostics was used to measure blood glucose levels. Lumbar spine and femur BMD were measured using dual‐energy x‐ray absorptiometry (DXA).

### 2.7 Cell culture

MIN6 cells (pancreatic β cells) were purchased from the American Type Culture Collection (Manassas, VA, USA) and cultured in low‐glucose DMEM with 5 mmol/L glucose, 100 U/mL penicillin, 0.1 mg/mL streptomycin, 5.5 mM 2‐mercaptoethanol and 10% horse serum.

### 2.8 Plasmid and transfection

Full‐length sequences of *PDIA6* and *SLC16A1* were synthesized and cloned into the pcDNA3.1‐CMV‐vector (Hanbio Biotechnology, Shanghai, China). Small interfering RNAs (siRNAs) precisely targeting *PDIA6* and *SLC16A1* (siPDIA6 and siSLC16A1), as well as siRNA control were synthesized by RiboBio (Guangzhou, China). Lipofectamine 3000 was used for transfection, as directed by the manufacturer.

### 2.9 Quantitative real‐time PCR (qRT‐PCR)

Total RNA was extracted from the samples using TRIzol reagent, and reverse transcription was performed on a T100 thermal cycler using the PrimeScript RT Reagent Kit. The SYBR Green qRT‐PCR Master Mix Kit was used in qRT‐PCR on a LightCycler 480 II. The relative gene expression levels were determined using the 2^−ΔΔCT^ method.

The specific primers used in the PCR analysis were as follows:

PDIA6, forward, 5′‐ AAGGCGAGTCTCCTGTGGATTATG‐3′ and reverse, 5′‐ ACGTCCTCTTGGCAATGTCCTC‐3′;
SLC16A1, forward, 5′‐ ATGCCACCACCAGCGAAGTG‐3′ and reverse, 5′‐ CAATCAAGCCACAGCCTGACAAG‐3′;
GAPDH, forward, 5′‐ATGATGACATCAAGAAGGTGG‐3′ and reverse, 5′‐TTGTCATACCAGGAAATGAGC‐3′.

### 2.10 Cell proliferation and cloning formation assays

MIN6 cells were inoculated into 96‐well plates at a density of 5 × 10^3^ cells/well, in three wells for each experimental group. The plates were then incubated for 48 h in a 5% CO_2_ incubator at 37°C, followed by addition of 10 mL of CCK‐8 liquid per well and incubation for 4 h at 37°C. The optical density (OD) at 450 nm was measured in each well using an ELISA reader. For the colony formation experiment, cells that have been transfected were placed in six‐well plates at 400 cells per well and cultured in DMEM medium with 10% FBS. The cells were kept in the medium for 2 weeks, with daily medium changes. After incubation, the cells were soaked in methanol and stained with 0.1% crystal violet. The colonies were then imaged and counted.

### 2.11 Cell apoptosis assay

Apoptosis rates were evaluated using the Annexin V‐FITC/PI staining kit (BD Bioscience, USA). After transfection, 1 × 10^4^ cells were treated with 3 μM OXA for 48 h. Cells were then selected and labelled for 15 min with Annexin V‐fluorescein isothiocyanate (FITC), followed by staining with propidium iodide (PI) for 5 min. The fraction of apoptotic cells was determined using a FACSCanto II flow cytometer.

### 2.12 Statistical analysis

The Venn diagram was constructed using the Jvenn software tool. The ceRNA network was visualized using Cytoscape.Each experiment was carried out thrice, and the findings are shown as mean ± standard deviation. SPSS 18.0 software (SPSS, Inc., USA) was utilized for the statistical analysis. The statistical analysis employed in this study involved utilizing the Student's *t*‐test to compare the two sample groups, while a one‐way ANOVA was employed to compare more than two groups. Pearson's correlation analysis was performed to ascertain the linear association between fold changes derived from RNA‐Seq and qPCR data. The same statistical method was used to calculate the correlation between genes and phenotypes. *p* < 0.05 was considered to indicate a statistically significant difference. We would like to express our gratitude to you for helping us improve the quality of our manuscript, and we appreciate your professionalism and seriousness.

## 3 RESULTS

### 3.1 GEO information

Based on the established criteria (please see Methods), four GEO data sets were selected: GSE15932, GSE148961, GSE62402 and GSE201543. Information on the data sets (including GSE numbers, detection systems, samples and RNA source type) is presented in Table 1. GSE15932 was matched with GSE62402 to generate the mRNA discovery cohort, whereas GSE148961 was paired with GSE201543 for miRNA analysis.

**TABLE 1:** Summary characteristics of the four GEO data sets from patients with type 2 diabetes (T2D) and osteoporosis (OP).

ID	GSE number	Platform	Samples	Source types	Disease	Group
1	GSE15932	GPL570	8 patients and 8 controls	Peripheral blood	T2D	mRNA
2	GSE148961	GPL25243	18 patients and 12 controls	Peripheral blood	T2D	miRNA
3	GSE62402	GPL5175	5 patients and 5 controls	Peripheral blood	OP	mRNA
4	GSE201543	GPL20712	6 patients and 4 controls	Peripheral blood	OP	miRNA

### 3.2 Identification and analysis of mRNAs in T2D

**FIGURE 1:**
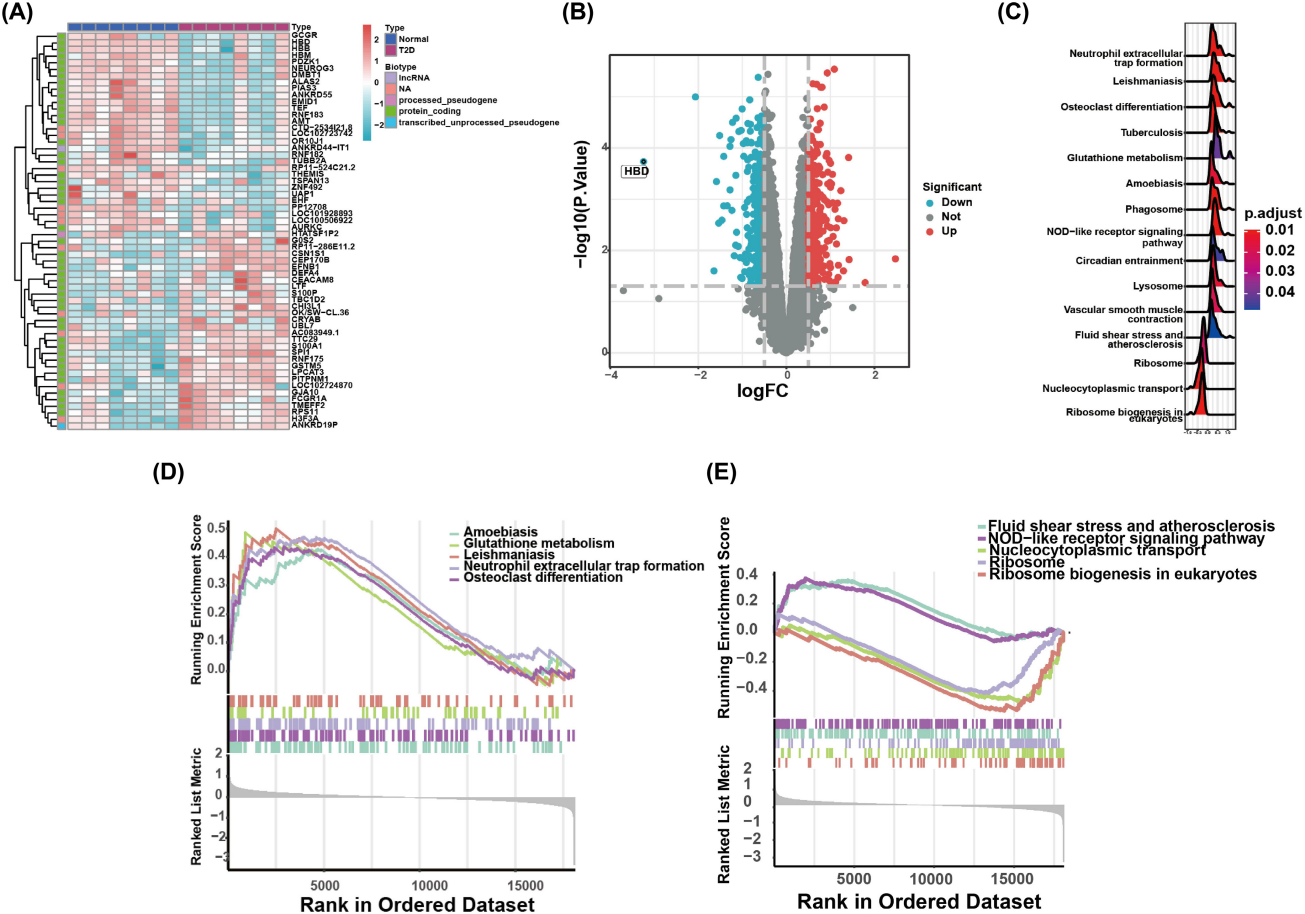
Differentially expressed mRNAs in T2D. (A) Heatmap of 60 differentially expressed mRNAs in GSE15932. (B) Volcano plot of differentially expressed miRNAs. Red, green and grey represent genes with upregulated, downregulated and non‐significantly altered expression, respectively. (C) Gene Ontology (GO) biological process analyses of 60 mRNA clusters in T2D. (D, E) Five terms indicate upregulated and downregulated biological functions.

Through the analysis of differentially expressed profiles between 8 normal and 8 T2D samples, 60 mRNAs were identified to be associated with T2D, shown in a heatmap and volcano plot (Figure 1A,B). Enrichment analysis revealed that these mRNAs are involved in various biological processes. Notably, three BP terms related to T2D were identified, namely ‘neutrophil extracellular trap formation’, ‘leishmaniasis’, and ‘osteoclast differentiation’ (Figure 1C). The BP terms for upregulated differentially expressed mRNAs included ‘amoebiasis’, ‘glutathione metabolism’, ‘leishmaniasis’, ‘neutrophil extracellular trap formation’ and ‘osteoclast differentiation’ (Figure 1D). Conversely, terms for downregulated differentially expressed mRNAs encompassed ‘fluid shear stress and atherosclerosis’, ‘NOD‐like receptor signalling pathway’, ‘nucleocytoplasmic transport’, ‘ribosome’ and ‘ribosome biogenesis in eukaryotes’ (Figure 1E).

### 3.3 Identification and analysis of mRNAs in OP

**FIGURE 2:**
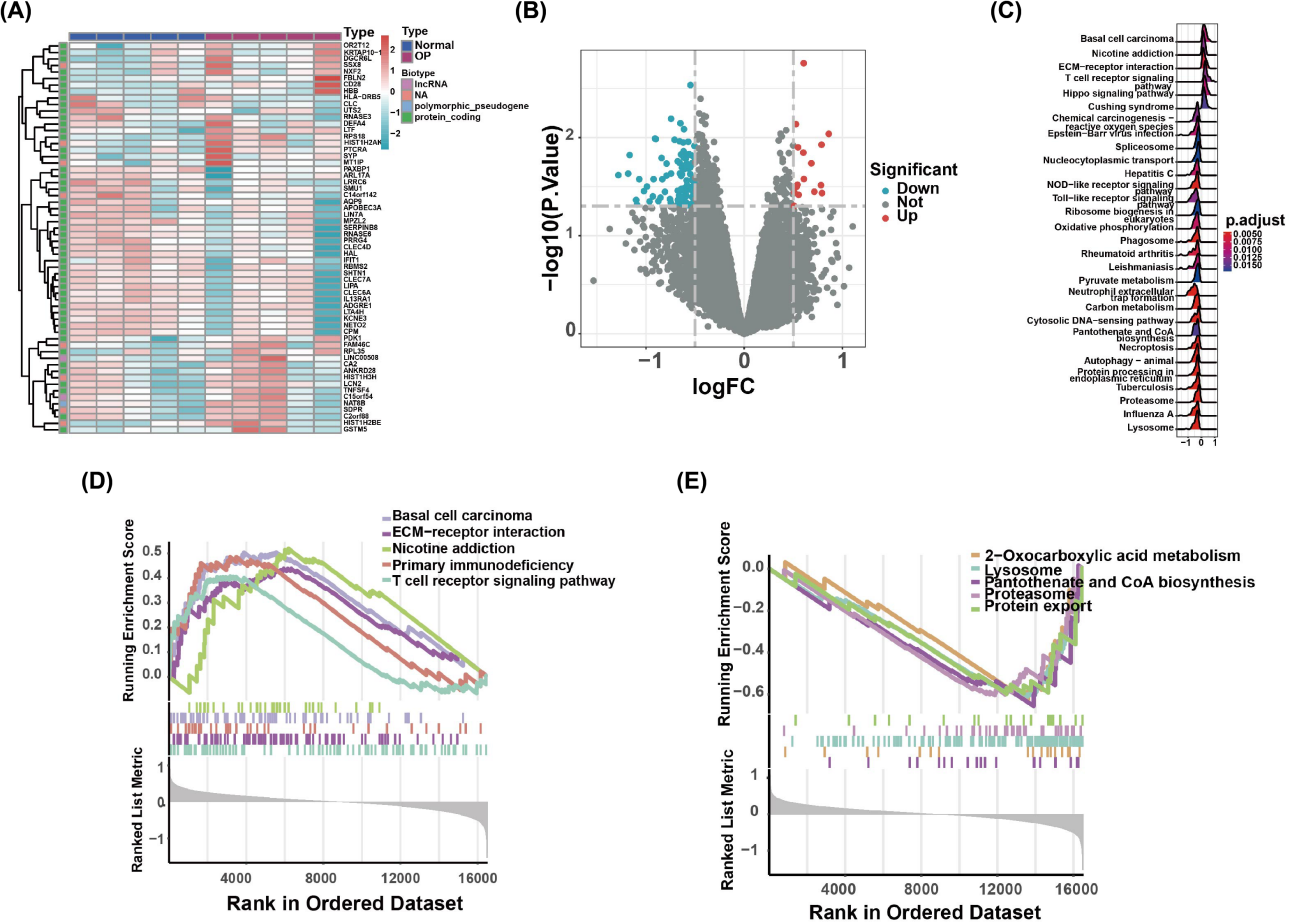
Differentially expressed mRNA in OP. (A) Heatmap of 60 differentially expressed mRNAs in GSE62402. (B) Volcano plot of differentially expressed miRNAs. Colour symbols as in Figure 1. (C) GO biological process analyses of 60 mRNA clusters in OP. (D, E) Five terms indicate upregulated and downregulated biological functions.

Through the analysis of the differentially expressed profiles between 8 normal and 8 OP samples, a set of 60 mRNAs was identified to be associated with OP. These findings are visualized in the heatmap and volcano plot in Figure 2A,B. Enrichment analysis revealed that these mRNAs are involved in multiple biological functions. The three prominent BP terms associated with OP were ‘basal cell carcinoma’, ‘nicotine addiction’ and ‘ECM–receptor interaction’ (Figure 2C). The BP terms related to upregulated differentially expressed mRNAs included ‘basal cell carcinoma’, ‘ECM–receptor interaction’, ‘nicotine addiction’, ‘primary immunodeficiency’ and ‘T‐cell receptor signalling pathway’ (Figure 2D). Conversely, downregulated differentially expressed mRNAs were enriched for ‘2‐oxocarboxylic acid metabolism’, ‘lysosome’, ‘pantothenate and CoA biosynthesis’, ‘proteasome’ and ‘protein export’ (Figure 2E).

### 3.4 Discovery cohort: Co‐expression modules in T2D and OP

**FIGURE 3:**
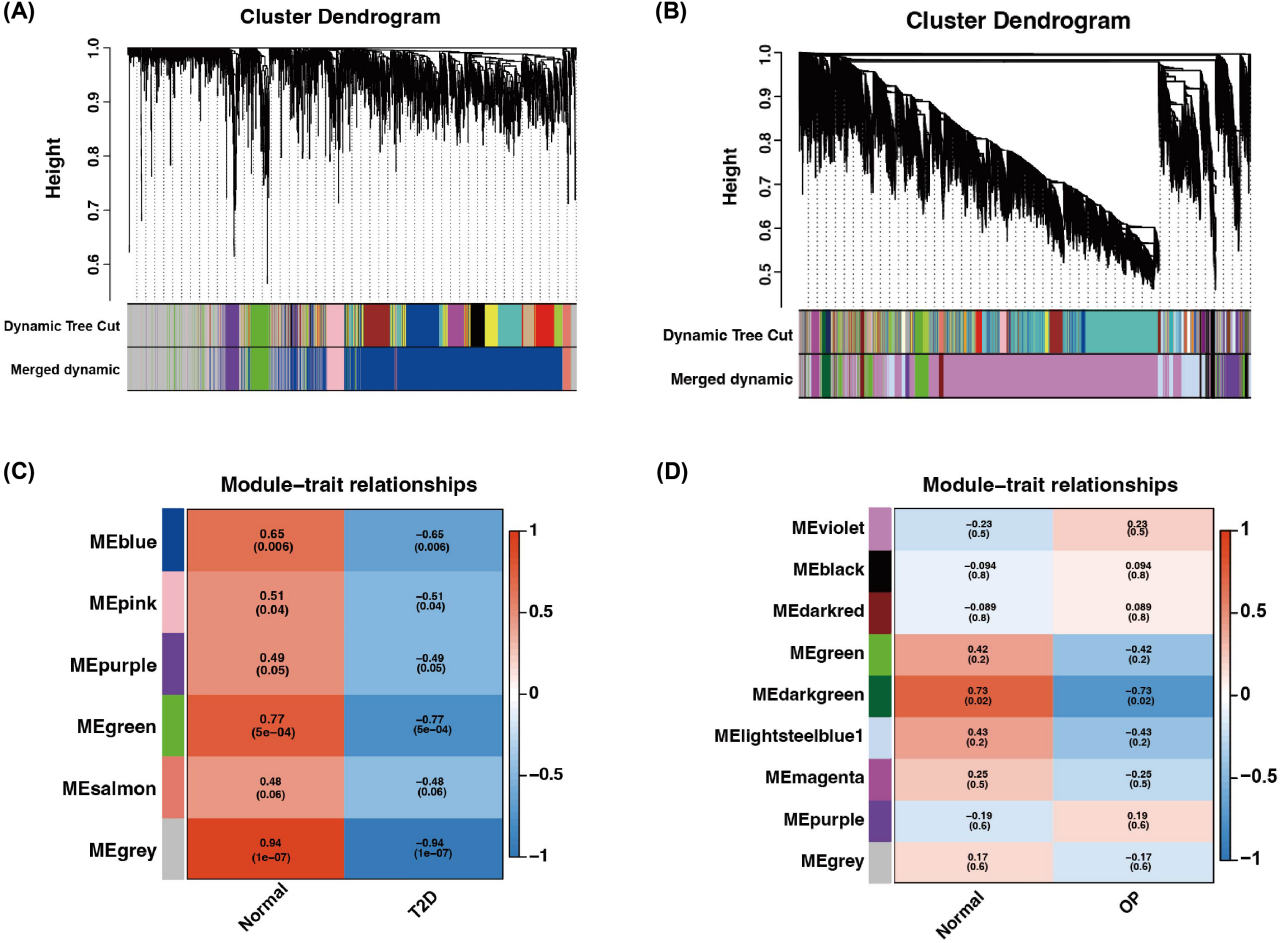
Weighted gene co‐expression network analysis. (A) Cluster dendrogram of co‐expressed genes in T2D. (B) Module–trait relationships in T2D. Each cell contains the corresponding correlation and *p* value. (C) Cluster dendrogram of co‐expressed genes in OP. (D) Module–trait relationships in OP. Each cell contains the corresponding correlation and *p* value.

By applying WGCNA to GSE15932, six modules were discovered, each represented by a distinct colour. Heatmaps of module–trait relationships were drawn based on Spearman correlation coefficients to assess the association between each module and disease (Figure 3A,B). Of these modules, the ‘blue’, ‘pink’, ‘green’ and ‘grey’ modules exhibited significant positive correlations with T2D (blue module: *r* = 0.006, *p* = 0.006; pink module: *r* = 0.04, *p* = 0.04; green module: *r* = 0.77, *p* = 5e−04) and were thus selected as T2D‐related modules. Similarly, nine modules were identified in GSE62402, with the ‘green’ module being the only one positively associated with OP (*r* = 0.73, *p* = 0.02) (Figure 3C,D).

### 3.5 Shared gene signatures in T2D and OP

**FIGURE 4:**
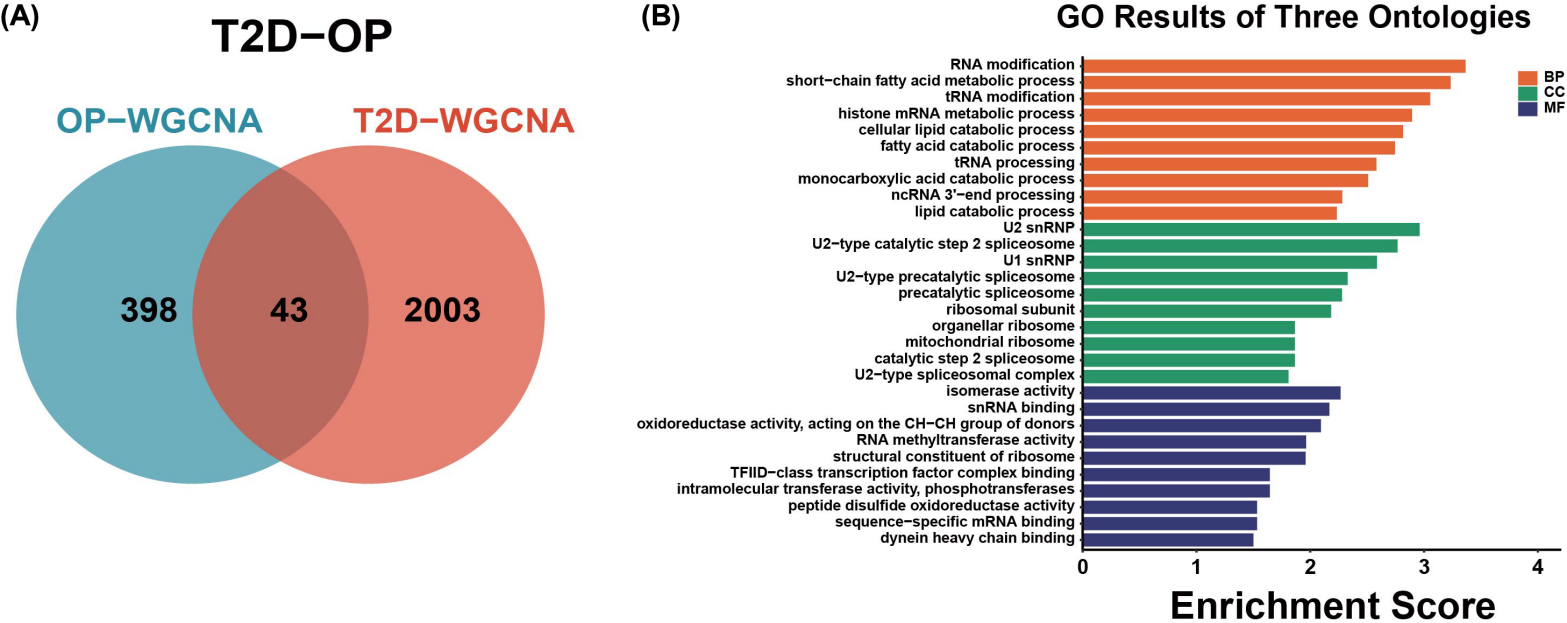
Identification of shared differentially expressed genes (DEGs). (A) Venn diagram showing the 43 genes at the intersection of the OP and T2D. (B) GO analyses using DAVID(v6.8) functional annotation analysis tool (https://david. ncifcrf.gov). BP, biological process, CC, cellular component; MF, molecular function.

A total of 43 genes overlapped in the modules positively correlated with T2D and OP and were defined as gene set 1 (GS1) (Figure 4A). These genes are significantly relevant to the pathogenesis of both diseases. To further investigate the potential functions of GS1, GO enrichment analysis was conducted. The analysis revealed that the top three significantly enriched GO BP, CC, and MF terms were ‘RNA modification’, ‘U2 snRNP’ and ‘isomerase activity’, respectively (Figure 4B). This finding confirms the crucial involvement of these components in both T2D and OP.

### 3.6 Unique gene signatures in T2D and OP

**FIGURE 5:**
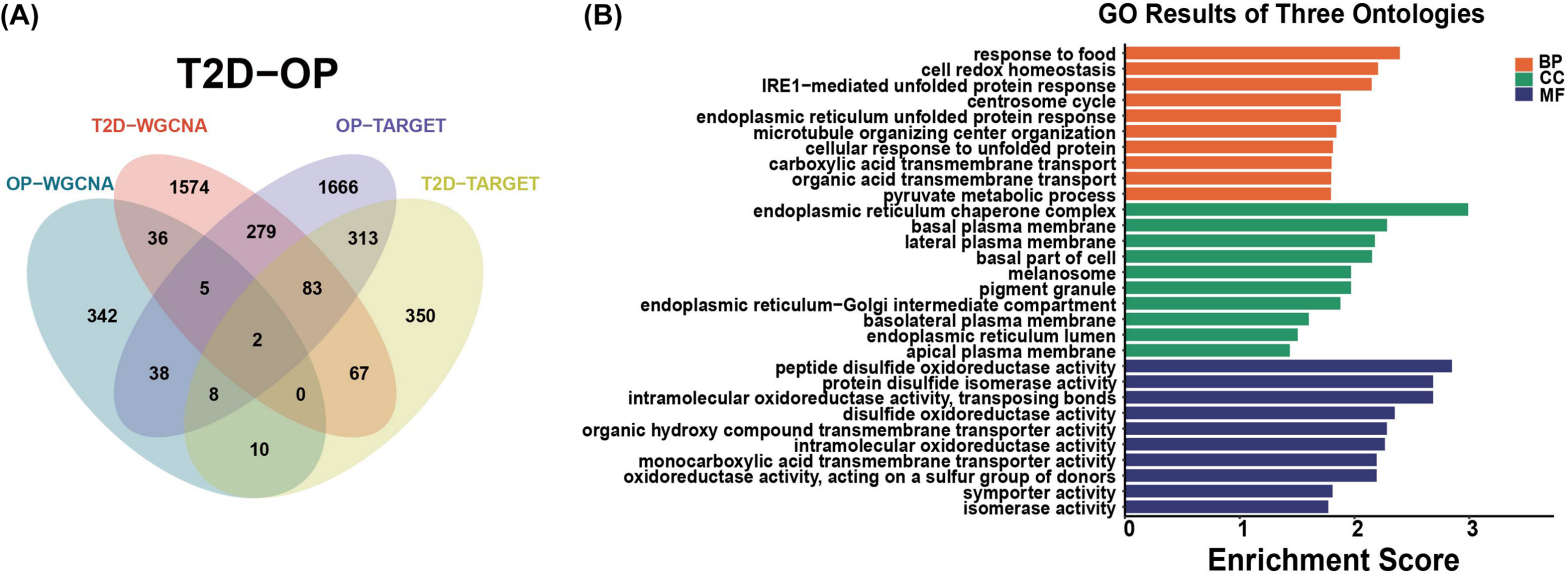
Identified DEGs. (A) Venn diagram showing the two DEGs identified using MCODE and TARGET analyses (intersection of OP and T2D). (B) GO analysis of DEGs using DAVID.

Four clusters were extracted using MCODE and TARGET analyses. Among these clusters, two genes, *PDIA6* and *SLC16A1*, were found to overlap between T2D and OP; these formed a gene set termed gene set 2 (GS2) (Figure 5A). To investigate potential functional links between these DEGs, DEGs were predominantly involved in processes including ‘response to food’, ‘endoplasmic reticulum chaperone complex’ and ‘peptide disulphide oxidoreductase activity’ (Figure 5B). The functional analysis of the DEGs is shown in Figure 5B.

### 3.7 Identification and analysis of miRNAs in T2D and OP

**FIGURE 6:**
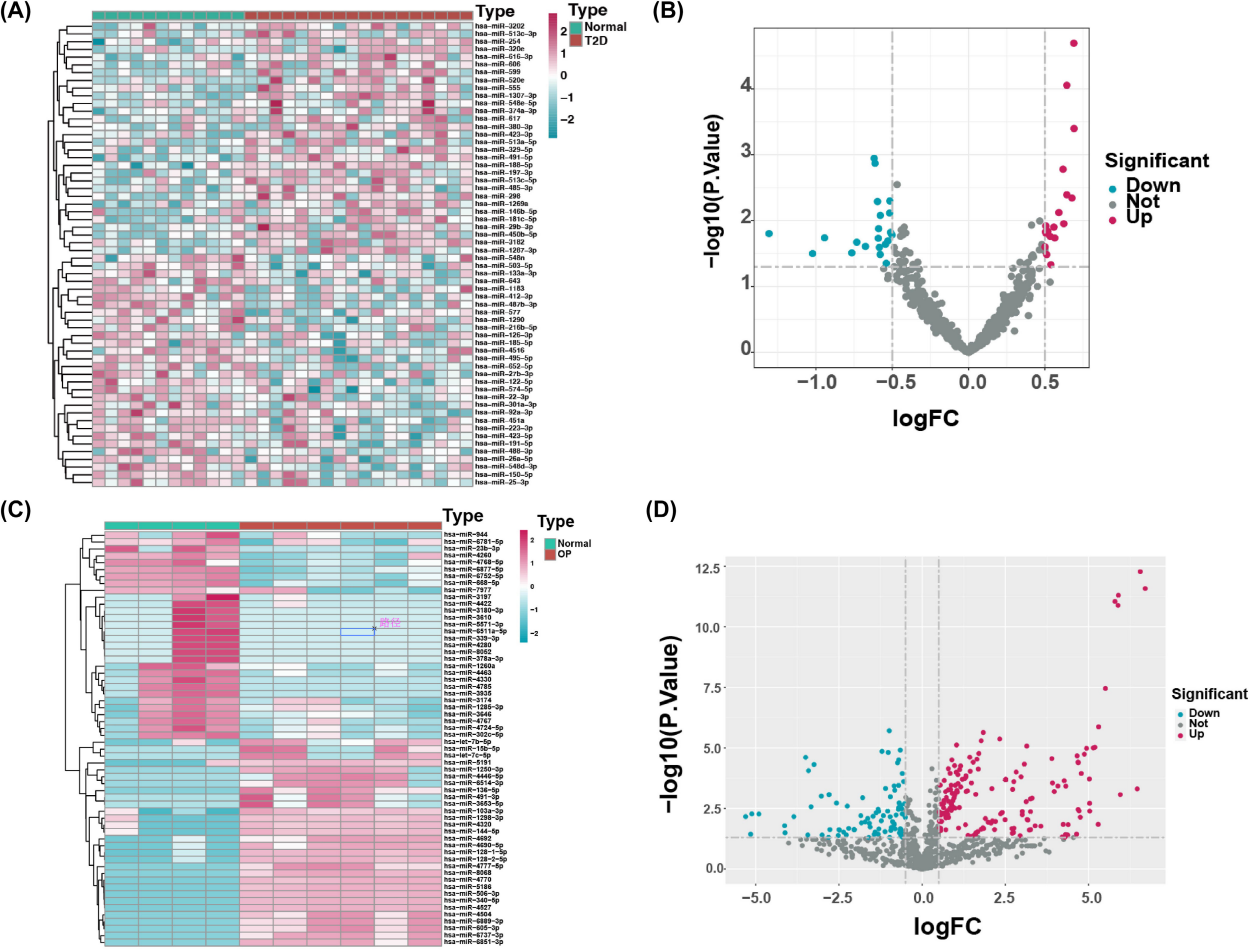
Differentially expressed miRNAs in T2D and OP. (A) Heatmap of 60 differentially expressed miRNAs in GSE15932. (B) Volcano plot of differentially expressed miRNAs. (C) Heatmap of 60 differentially expressed miRNAs in GSE62402. (D) Volcano plot of differentially expressed miRNAs.

Sixty miRNAs were associated with T2D by evaluating differentially expressed profiles between normal and T2D samples. A heatmap and a volcano plot (Figure 6A,B) show the expression patterns of these miRNAs. Similarly, through analysis of differentially expressed profiles between normal and OP samples, another set of 60 miRNAs was identified to be associated with T2D. A heatmap and a volcano plot (Figure 6C,D) illustrate the expression patterns of these miRNAs.

### 3.8 Identification and analysis of shared miRNA–target gene network in T2D and OP

**FIGURE 7:**
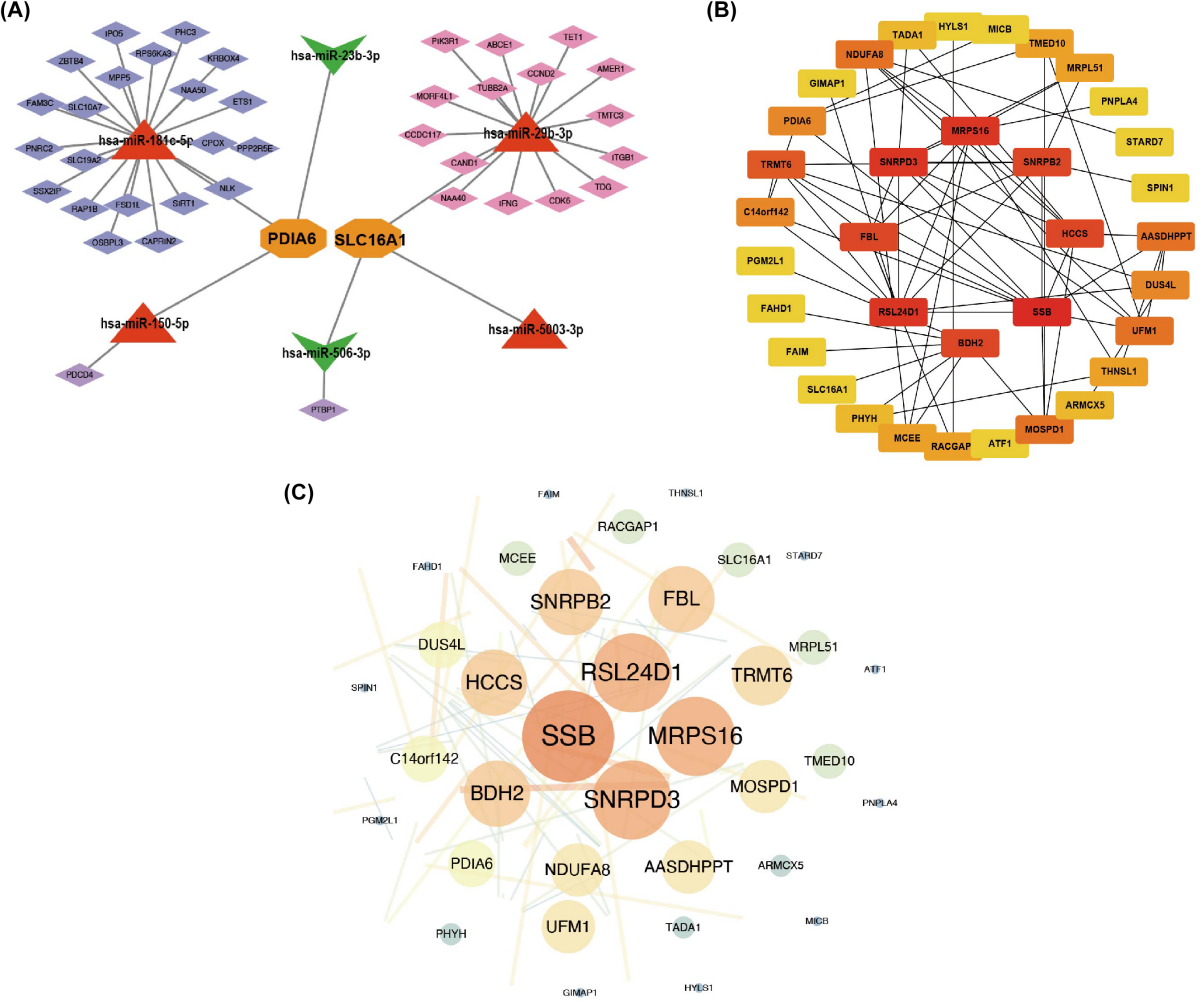
miRNA–target gene regulatory network. (A) Competitive endogenous RNA (ceRNA) network analysis of miRNAs–target genes. Red triangles represent miRNAs and orange represent overlapped shared genes. (B, C) Protein–protein interaction (PPI) analysis (B) and hub genes (C) of related DEGs.

Based on the GEO database, we obtained data regarding the expression of these shared miRNAs in the disorders. Accordingly, the expression of 18 miRNAs was upregulated, whereas that of 21 miRNAs was downregulated in T2D. For OP, 183 miRNAs showed upregulated, while 86 miRNAs showed downregulated expression. Notably, six miRNAs overlapped between T2D and OP: hsa‐miR‐181c‐5p, hsa‐miR‐29b‐3p, hsa‐miR‐150‐5p and hsa‐miR‐5003‐3p. In T2D, 833 mRNAs were targeted by miRNAs, while, in OP, the number rose to 2394. Among the six overlapping miRNAs, the two genes, *PDIA6* and *SLC16A1*, were targeted. Consequently, we constructed a comprehensive miRNA–mRNA network that consisted of the six miRNAs and 38 mRNAs (Figure 7A), as well as an interaction diagram to illustrate the relationships among 35 targeted genes (Figure 7B). Finally, a PPI network was established to analyse the 35 targeted genes (Figure 7C).

### 3.9 Expression of *PDIA6* and *SLC16A1* is markedly downregulated in patients with T2DOP

**FIGURE 8:**
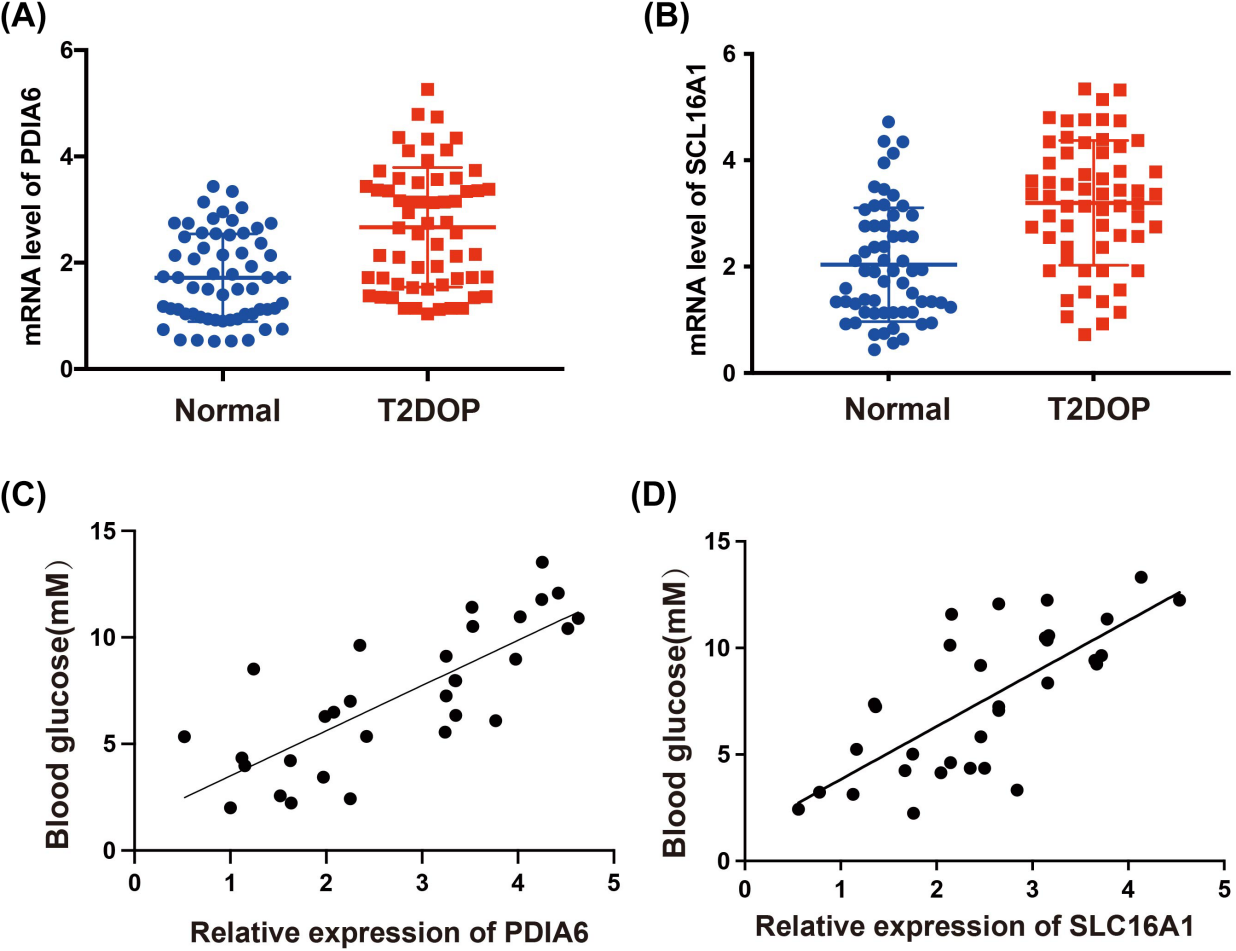
*PDIA6* and *SLC16A1* expression is significantly upregulated in patients with T2D. (A) The mRNA levels of *PDIA6* and *SLC16A1* in patients with diabetes and healthy volunteers were detected using reverse transcription quantitative polymerase chain reaction analysis. (B) Pearson's correlation analysis was performed to determine the association between *PDIA6* and *SLC16A1* expression and blood glucose levels in patients with T2D.

To investigate the significance of *PDIA6* and *SLC16A1* in diabetes, we examined their expression in blood specimens obtained from 60 patients with T2DOPand 60 healthy controls. qRT‐PCR analysis indicated significant upregulation of *PDIA6* and *SLC16A1* mRNA levels in individuals with diabetes when compared to healthy volunteers (Figure 8A,B). Moreover, we established that a positive interaction exists between *PDIA6* and *SLC16A1*expression and blood glucose levels (Figure 8C,D). We subsequently explored the relationship between patient clinicopathological characteristics and the expression of *PDIA6* and *SLC16A1* and revealed a significant association between high *PDIA6* expression and factors such as age, blood glucose levels, body mass index (BMI), and 25‐hydroxyvitamin D [25(OH)D] (Table 2). Additionally, high *SLC16A1* expression was found to be associated with blood glucose levels and 25(OH)D (Table 3). Obese T2DM patients are more susceptible to insulin resistance, elevated blood glucose levels, and lipid disorders. Further, there is a negative correlation between 25(OH)D concentration and the development of T2DM.A higher level of 25(OH)D is associated with a reduced risk of T2DM. The results showed that most patients with high *PDIA6* and *SLC16A1* expression had elevated blood glucose and decreased 25(OH)D levels, and the levels of *PDIA6* and *SLC16A1* may be related to insulin secretion, islet beta cell function and insulin resistance.

**TABLE 2:** Association between *PDIA6* expression and clinicopathologic features of 60 patients with T2DOP.

Characteristics	*PDIA6* expression		*χ* ^2^‐value	*p*‐value
	High (*n* = 30)	Low (*n* = 30)		
Age, years				
≥40	20	9	8.076	0.004*
<40	10	21		
Gender				
Male	18	16	0.271	0.602
Female	12	14		
Blood glucose level				
≥7.0 mM	22	11	8.148	0.004*
<7.0 mM	8	19		
BMD (g/cm^2^)				
≥−2.5	7	14	3.590	0.058
<−2.5	23	16		
BMI(kg/m^2^)				
≥24	21	13	4.344	0.037*
<24	9	17		
25(OH) D (μg/L)				
≥30	11	21	6.696	0.010*
<30	19	9		

*Note*: Chi‐squared test was used to test the association between two categorical variables.

* Statistically significant.

**TABLE 3:** Association between *SLC16A1* expression and clinicopathologic features of 60 patients with T2DOP.

Characteristics	SLC16A1 expression		*χ* ^2^‐value	*p*‐value
	High (*n* = 30)	Low (*n* = 30)		
Age, years				
≥40	24	19	2.052	0.152
<40	6	11		
Gender				
Male	23	18	1.926	0.165
Female	7	12		
Blood glucose level				
≥7.0 mM	20	12	4.286	0.038*
<7.0 mM	10	18		
BMD (g/cm^2^)				
≥ − 2.5	11	15	1.086	0.297
<−2.5	19	15		
BMI(kg/m^2^)				
≥24	20	17	0.635	0.426
<24	10	13		
25(OH) D (μg/L)				
≥30	13	22	5.554	0.018*
<30	17	8		

*Note*: Chi‐squared test was used to test the association between two categorical variables.

* Statistically significant.

### 3.10 *PDIA6* and *SLC16A1* suppress insulin secretion and the proliferation of pancreatic β‐cell lines

**FIGURE 9:**
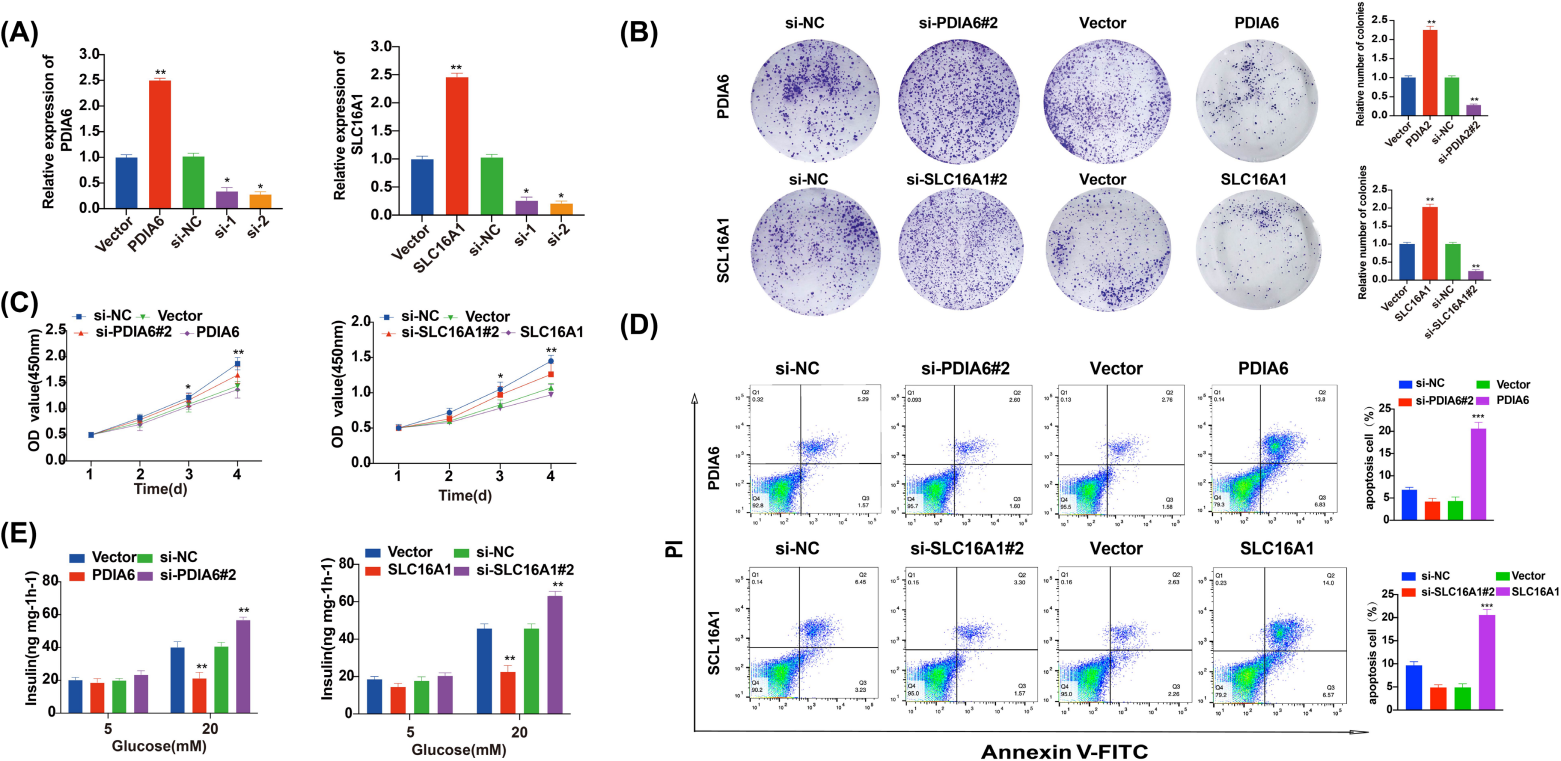
*PDIA6* and *SLC16A1* suppress insulin secretion and pancreatic β‐cell proliferation. (A) The mRNA levels of *PDIA6* and *SLC16A1* were detected using reverse transcription quantitative polymerase chain reaction analysis. (B) A colony formation assay and (C) CCK‐8 assay were performed to assess the effect of *PDIA6* and *SLC16A1* on cell proliferation. (D) Flow cytometry was performed to assess the effect of *PDIA6* and *SLC16A1* on cell apoptosis. (E) Transfected MIN6 cells were incubated with 5 mM or 20 mM glucose for 90 min and the rate of insulin secretion was detected in each group. Data are shown as the mean ± standard deviation of three independent experiments. *p < 0.05, **p < 0.01 and ***p < 0.001.

To elucidate the functional role of these genes, we overexpressed or silenced *PDIA6* and *SLC16A1* in MIN6 cells. Following transfection with *PDIA6* and *SLC16A1* plasmid or siPDIA6 and siSLC16A1, *PDIA6* and *SLC16A1* expression was extremely upregulated or downregulated, respectively, compared to that in their respective control groups (Figure 9A). Notably, siPDIA6#2 and siSLC16A1#2 exhibited superior efficiency compared to siPDIA6#1 and siSLC16A1#1 and were thus utilized for subsequent experiments. Overexpression of *PDIA6* and *SLC16A1* significantly inhibited the formation of MIN6 cell colonies, whereas inhibition of *PDIA6* and *SLC16A1* expression reversed this effect (Figure 9B). Additionally, the CCK‐8 assay revealed that upregulation of the expression of *PDIA6* and *SLC16A1* significantly inhibited the proliferation of MIN6 cells compared to the controls, while inhibition of *PDIA6* and *SLC16A1* expression demonstrated the opposite effect (Figure 9C). Flow cytometry analysis indicated that overexpression of *PDIA6* and *SLC16A1* increased the apoptosis ratio of MIN6 cells, whereas knockdown of *PDIA6* and *SLC16A1* resulted in contrasting effects (Figure 9D). Lastly, we observed that overexpression of *PDIA6* and *SLC16A1* significantly reduced insulin secretion levels in MIN6 cells upon exposure to glucose, in comparison to the control group. Conversely, inhibition of *PDIA6* and *SLC16A1* expression significantly increased insulin secretion (Figure 9E). Thus, the results suggest that downregulation of *PDIA6* and *SLC16A1* expression facilitates insulin secretion and proliferation of pancreatic β‐cell lines.

## 4 DISCUSSION

T2D and OP are long‐term conditions that impair glucose and bone metabolism, and pose a significant health burden, particularly among the older population.^14^ T2D and OP share several risk factors, including non‐modifiable factors, such as age and genetic susceptibility, and modifiable factors, such as lifestyle and nutrition. Consequently, both diseases commonly coexist. The prevalence of diabetes has gradually increased due to increased life expectancy, with T2D accounting for over 90% of all diabetes cases globally. Diabetes can have macrovascular consequences, such as stroke and heart disease, as well as microvascular complications, such as nephropathy. These complications contribute significantly to increased morbidity and mortality rates.^15^ OP is a common disorder characterized by low bone mass and microarchitectural degeneration of bone tissue, resulting in bone fragility and increased fracture risk. The disorder affects both men and women, although postmenopausal women are particularly at higher risk.^16^

The pathogenesis of T2D remains unclear, but previous studies have documented co‐occurrence of T2D and OP. Epidemiological observations have shown that individuals with T2D have a higher risk of fragility fractures than the general non‐diabetic population.^17^ The mechanisms underlying bone fractured in T2D are diverse and unresolved.^18^ According to previous studies, hyperglycaemia, inflammation, oxidative stress, and changes in osteoblast function play important roles in the development of OP in the setting of T2D. Hyperglycaemia and the formation of advanced glycation end products (AGEs) are two factors that may contribute to decreased bone strength.^19^ This is the first study to apply WGCNA to investigate shared mechanisms between T2D and OP. WGCNA is an algorithm that enables the identification of co‐expression gene modules with high biological significance associated with clinical traits. Among the co‐expressed gene modules identified in T2D and OP, enrichment was observed in modules that involve RNA modifications, cellular lipid and fatty acid catabolism, and isomerase activity. Hyperglycaemia/hyperinsulinaemia and disrupted fatty acid metabolism in patients with diabetes promote enhanced oxidative processes, resulting in the production of free radicals and other reactive oxygen species (ROS).^20^ This increased oxidative stress is associated with an elevated risk of bone fragility and fractures.^21^

We used the GEO database and miRTarbase to construct a shared miRNA–gene network.^22^ Among the identified miRNAs, we detected six miRNAs that overlapped between T2D and OP, including hsa‐miR‐181c‐5p, hsa‐miR‐29b‐3p, hsa‐miR‐150‐5p, and hsa‐miR‐5003‐3p. Two genes (*PDIA6* and *SLC16A1*) were co‐expressed in T2D and OP and miRNA–mRNA networks were constructed. *PDIA6* is a member of the protein disulphide isomerase (PDI) family, which consists of oxidoreductases that catalyse the creation and rearrangement of disulphide bonds during protein folding. *PDIA6* has been implicated in β‐cell dysfunction and diabetes, although its precise function in β cells in vivo remains poorly understood. *SLC16A1* is expressed in various tissues, including the liver,^23^ gastric epithelium and intestine.^24^, ^25^ *SLC16A1* is responsible for the transport of monocarboxylic acid metabolites, such as lactate, pyruvate and ketone bodies,^26^ which are involved in the formation of mitochondrial lactate oxidation complexes.^27^ The role of *SLC16A1* in glucose metabolism and its function in β cells are not yet clear.

Our subsequent investigations confirmed that expression of *PDIA6* and *SLC16A1* in patients with T2DOP was upregulated and that their expression correlated with elevated blood glucose levels. Furthermore, the expression of *PDIA6* and *SLC16A1* was associated with patient factors such as blood glucose, BMI and 25(OH)D levels. These associations may be linked to changes in the expression levels of certain proteins with increasing body age, resulting in increased blood sugar levels, higher BMI, and development of OP and greater expression of *PDIA6* and SLC16A1. Functional experiments, including colony formation, CCK‐8 and apoptosis assays, indicated that the inhibition of *PDIA6* and *SLC16A1* facilitated proliferation of pancreatic β‐cells, suppressed apoptosis, and enhanced insulin secretion. This study postulates that various factors, such as the cellular microenvironment, metabolism and activation of oncogenes, may contribute to the divergent functions of *PDIA6* and *SLC16A1*.

Finally, we investigated the functional involvement of *PDIA6* and *SLC16A1* in T2D and revealed the underlying mechanisms involved in insulin secretion and pancreatic cell activity. The findings suggest that *PDIA6* and *SLC16A1* are involved in the pathophysiology of T2D and OP, and could serve as innovative biomarkers for the recognition of these disorders.

## AUTHOR CONTRIBUTIONS

**Ashuai Du:** Conceptualization (equal). **Rong Xu:** Data curation (equal); formal analysis (equal). **Qinglong Yang:** Investigation (equal); methodology (equal). **Yingxue Lu:** Resources (equal); software (equal). **Xinhua Luo:** Funding acquisition (equal); project administration (equal); writing – review and editing (equal).

## CONFLICT OF INTEREST STATEMENT

The authors confirm that there are no conflicts of interest.

## Data Availability

The data supporting the findings of this research are available from the corresponding author upon request.
